# Modified single-site two-port laparoscopic appendectomy with single-instrument knotting and absorbable clips in children: a retrospective study

**DOI:** 10.3389/fped.2026.1774339

**Published:** 2026-05-15

**Authors:** Yuan-fei He, Yong Yang, Shi-qin Qi, Cheng-xiao Zhou, Tao Zhang

**Affiliations:** Department of Pediatric Surgery, Anhui Provincal Children’s Hospital, He Fei, Anhui, China

**Keywords:** absorbable clips, appendectomy, Hem-o-lok clips, postoperative complications, single-instrument knotting, single-site laparoscopy

## Abstract

**Objective:**

To evaluate the clinical efficacy of a modified single-site two-port laparoscopic suspension technique combined with single-instrument knotting and absorbable clips in pediatric appendectomy, and to compare it with the single-site Hem-o-lok clip technique.

**Methods:**

This retrospective study reviewed pediatric patients with acute appendicitis treated at our institution between January 2024 and March 2025. Patients who underwent one of the two single-site two-port laparoscopic techniques were included and classified according to the closure method used for the appendiceal stump and mesoappendix: absorbable clips combined with single-instrument knotting or Hem-o-lok clips. Perioperative outcomes, postoperative complications, ultrasonographic findings, and postoperative inflammatory markers were evaluated.

**Results:**

A total of 133 pediatric patients were included, with 70 in the absorbable-clip group and 63 in the Hem-o-lok group. No significant differences were found between the two groups in abdominal drain placement, time to drain removal, intraoperative blood loss, time to first postoperative flatus, or postoperative hospital stay (*P* > 0.05). Operative time was significantly shorter in the Hem-o-lok group (*P* < 0.05). The incidences of subacute intestinal obstruction and postoperative abdominal pain were significantly higher in the Hem-o-lok group (*P* < 0.05), whereas incision infection, stump inflammation, and fecal leakage did not differ significantly between groups (*P* > 0.05). Ultrasonographic follow-up and postoperative inflammatory markers showed a stronger and more sustained inflammatory response in the Hem-o-lok group during follow-up. Two patients in the Hem-o-lok group required reoperation for clip removal because of refractory symptoms.

**Conclusion:**

Although the modified single-site two-port laparoscopic suspension appendectomy combined with single-instrument knotting and absorbable clips required a slightly longer operative time than the Hem-o-lok clip technique, it was associated with fewer postoperative complications and a milder postoperative inflammatory response. These findings should be interpreted cautiously and confirmed in larger prospective studies with longer follow-up.

## 1 Introduction

Acute appendicitis is one of the most common causes of acute abdomen in children (1). It can occur at any age, with a marked increase in incidence among children older than 5 years(2). Appendicolith obstruction, bacterial translocation, and anatomical factors are major contributors to its development (3). In pediatric patients, the disease often progresses rapidly and is prone to perforation; therefore, early surgical removal remains the optimal treatment strategy (4).

Laparoscopic appendectomy has become the preferred surgical approach for acute appendicitis. Owing to its minimal invasiveness and superior cosmetic outcomes, single-site laparoscopic techniques have been increasingly adopted in recent years (5). At present, Hem-o-lok clips are commonly used for closure of the appendiceal stump and mesoappendix during laparoscopic surgery. However, as non-absorbable foreign materials, they may induce local inflammatory reactions and postoperative adhesions (6).

The modified single-site two-port laparoscopic suspension technique optimizes trocar placement and suspension fixation, resulting in smaller umbilical incisions and improved cosmetic outcomes(7). The suspension provides adequate operative space, and when combined with single-instrument knotting and absorbable clips, it ensures secure closure while minimizing foreign-body retention(8). This study compared the clinical outcomes of this modified technique with those of the Hem-o-lok clip method, with particular focus on the advantages of single-instrument knotting combined with absorbable clips in reducing postoperative complications and attenuating tissue reactions, thereby providing clinical evidence for further optimization of single-site laparoscopic appendectomy.

## 2 Materials and methods

### 2.1 Retrospective analysis

Pediatric patients with acute appendicitis who met the predefined inclusion criteria and underwent one of the two single-site two-port laparoscopic techniques under comparison at our institution between January 2024 and March 2025 were retrospectively identified. Other approaches to pediatric appendicitis, such as conventional multiport laparoscopy, open appendectomy, or endoscopic retrograde appendiceal therapy (ERAT), were also used during the same period, but these patients were not included in this comparative analysis. The included patients were classified according to the technique used for appendiceal stump and mesoappendix management. The surgical technique was selected according to routine clinical practice, mainly based on surgeon preference, device availability, and intraoperative local conditions, with potential selection bias and indication bias not being completely excluded.

### 2.2 Inclusion and exclusion criteria

Inclusion criteria: (1) patients younger than 18 years with a definitive diagnosis of acute appendicitis confirmed by intraoperative findings and postoperative pathological examination; (2) patients who underwent appendectomy in the Department of General Surgery at our institution; (3) first episode of appendicitis; (4) complete clinical data available for analysis.

Exclusion criteria: (1) pathological findings inconsistent with acute appendicitis; (2) patients with poor cardiopulmonary function or concomitant systemic diseases, including hepatic or renal disorders; (3) incomplete clinical data; (4) patients who underwent open appendectomy or ERAT.

### 2.3 Surgical methods

**Figure 1:**
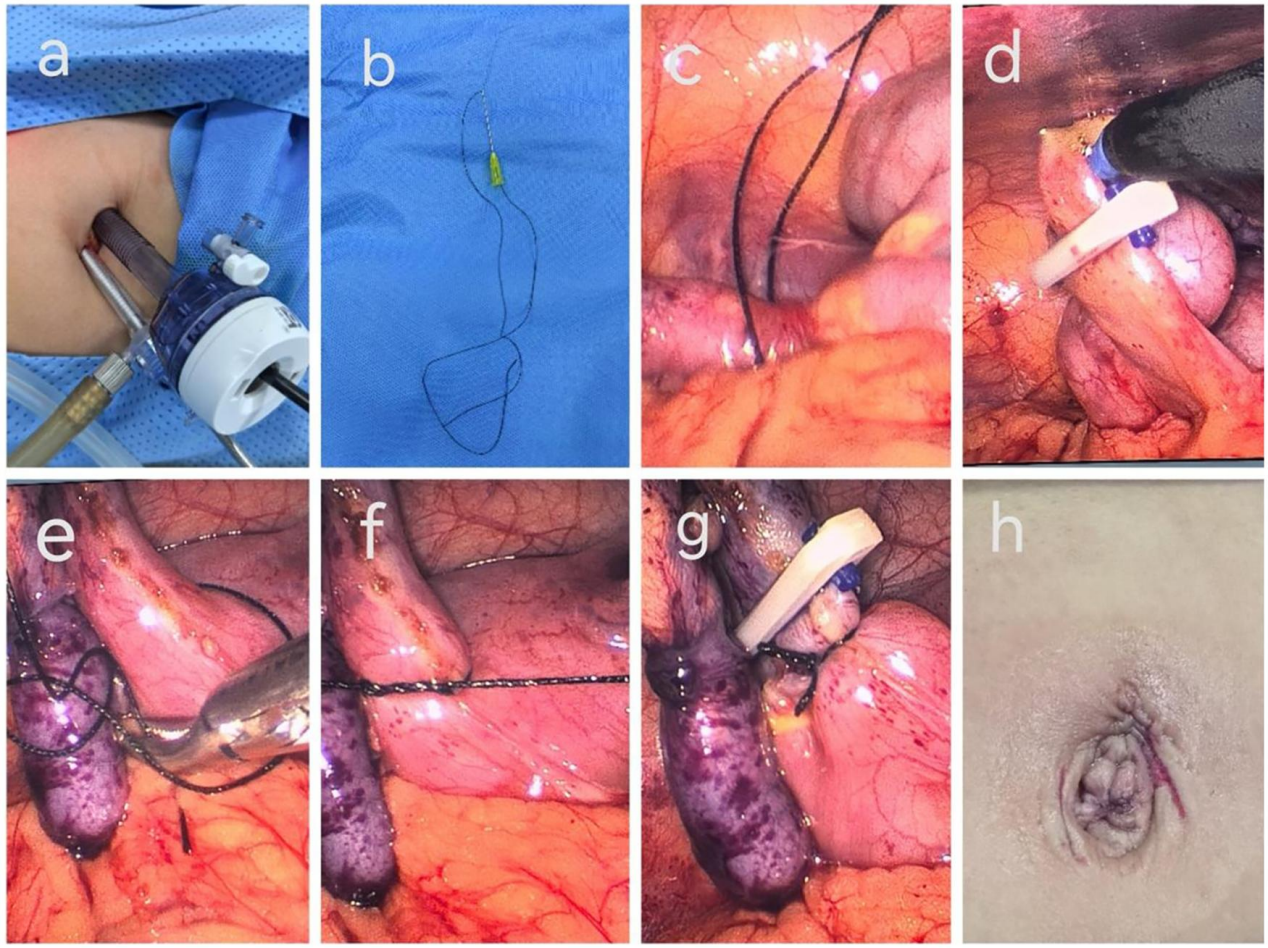
Intraoperative images of a pediatric patient undergoing modified single-incision two-port laparoscopic suspension appendectomy combined with single-instrument knotting and absorbable clip closure. **(a)** Schematic illustration of the single-incision two-port umbilical layout; **(b)** A No. 9 needle loaded with No. 7 silk suture; **(c)** Suspension of the appendix; **(d)** Absorbable clip closure of the mesoappendix; **(e)** Single-instrument looping of the suture; **(f)** Single-instrument knot tying; **(g)** Absorbable clip closure of the appendiceal stump; **(h)** Postoperative appearance of the umbilical incision.

2.3.1 *Modified single-site two-port laparoscopic suspension appendectomy combined with single-instrument knotting and absorbable clips*: Patients were fasted for 6–8 hours preoperatively and received intravenous third-generation cephalosporin antibiotics. They were positioned in Trendelenburg with left lateral tilt, and the surgeon stood on the patient's left side. After anesthesia, a 0.5 cm incision was made at the lower left umbilicus for a 5 mm trocar to establish pneumoperitoneum. A 1.0 cm incision was made at the upper right umbilicus for a 10-mm trocar for the laparoscope and forceps insertion (Figure 1a). The appendix was located by tracing the taeniae coli. Excess fluid was removed with negative-pressure suction. The appendix was suspended using a No. 9 needle and No. 7 silk suture (Figures 1b,c). Absorbable clips were applied to the mesoappendix, which was dissected using an electrocautery hook (Figure 1d). The appendix base was ligated with single-instrument knotting (Figures 1e,f), and a 10 mm absorbable clip was applied proximal to the ligation (Figure 1g). The appendix was transected above the clip and removed through the 10 mm trocar. The stump was cauterized without inversion. Drain placement was based on intraoperative findings. After confirming instrument and gauze counts, the abdominal wall was closed using absorbable sutures, with cosmetic reconstruction of the umbilical incision and sealing with tissue adhesive (Figure 1h). The resected specimen was sent for pathological examination. Postoperatively, patients were kept nil per os, and oral intake resumed after bowel function recovery and passage of flatus. Postoperative care included anti-infective therapy and fluid supplementation.

**Figure 2:**
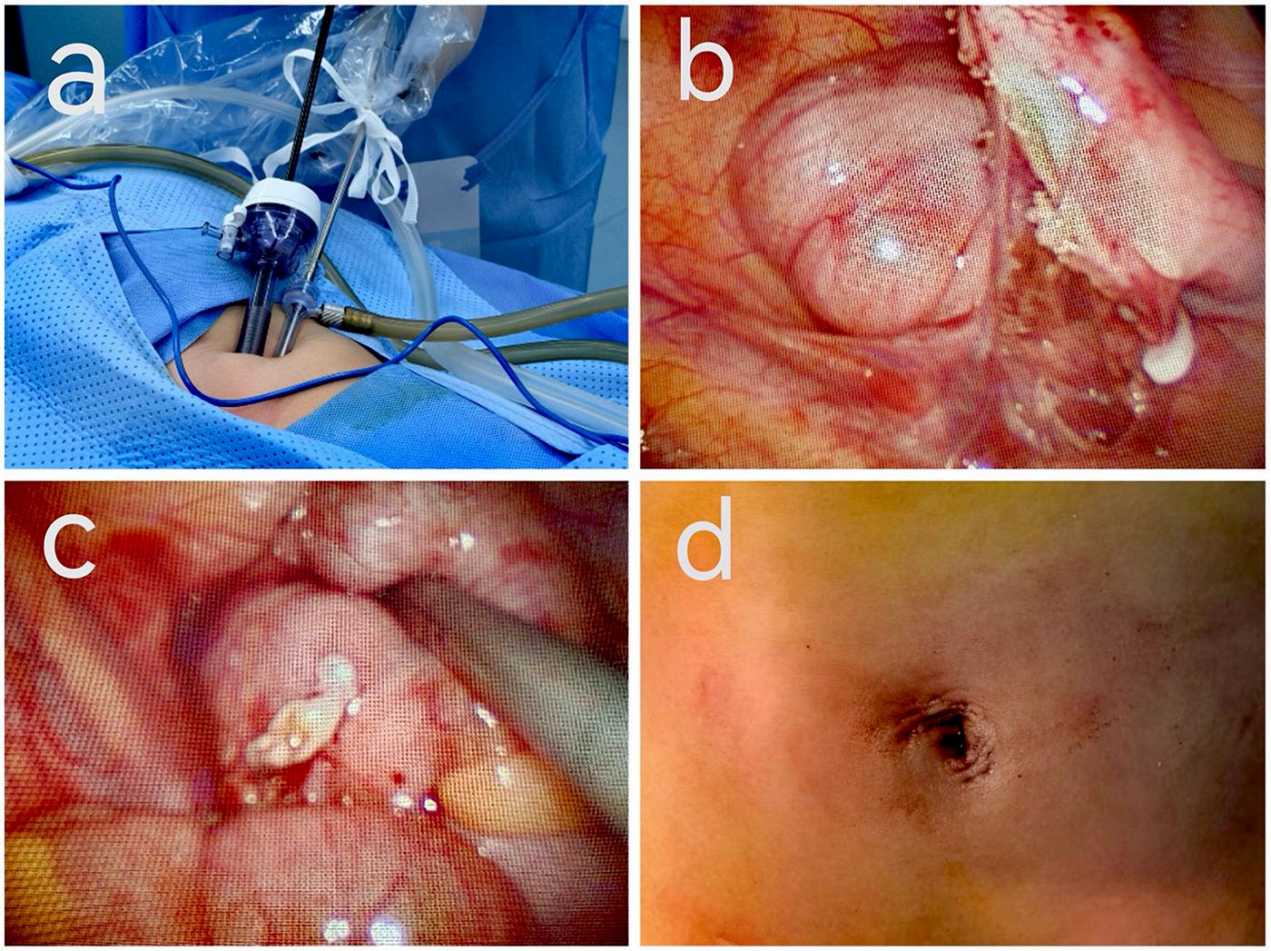
Intraoperative images of a pediatric patient undergoing single-incision two-port laparoscopic suspension appendectomy combined with Hem-o-lok clip closure. **(a)** Schematic illustration of the single-incision two-port umbilical layout and operative setup; **(b)** Hem-o-lok clip closure of the mesoappendix; **(c)** Hem-o-lok clip closure of the appendiceal stump; **(d)** Postoperative appearance of the umbilical incision.

2.3.2 *Single-site two-port laparoscopic suspension appendectomy using Hem-o-lok clips*: After induction of stable anesthesia, trocars were inserted and the appendix was suspended using the same approach as described above (Figure 2a). Hem-o-lok clips were applied to occlude the mesoappendix and the appendiceal base (Figures 2b,c).The appendix was resected using the same technique. After specimen retrieval, the appendiceal stump was cauterized with an electrocautery hook without stump inversion. Placement of a drainage tube was determined based on intraoperative findings. The abdominal wall was closed in layers with absorbable sutures, the umbilical incision was cosmetically reconstructed, and the skin was sealed using medical-grade biological tissue adhesive (Figure 2d).

**Figure 3:**
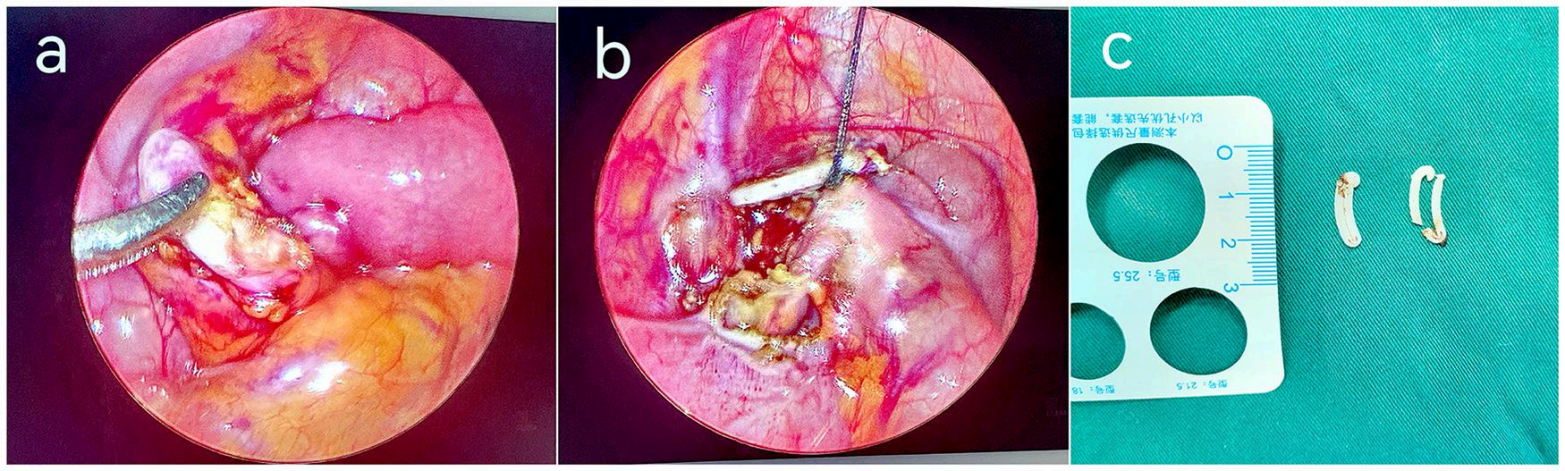
Intraoperative images showing laparoscopic removal of a Hem-o-lok clip. **(a)** Dense adhesions surrounding the Hem-o-lok clip at the ileocecal region; **(b)** Dissection and exposure of the Hem-o-lok clip; **(c)** Successful removal of the Hem-o-lok clip.

2.3.3 *Laparoscopic exploration and intraperitoneal foreign-body removal:* For patients requiring reoperation, laparoscopic exploration was performed as follows: after induction of stable anesthesia, a left periumbilical incision was made for a 5-mm trocar to establish pneumoperitoneum, and a 10-mm trocar was inserted at the right periumbilical site. Another 5-mm trocar was placed in the left lower abdomen for grasping forceps. Under laparoscopic visualization, adhesions at the site of the Hem-o-lok clips were identified (Figure 3a). The clips were dissected, cut, and removed extracorporeally (Figures 3b, c). No active bleeding was observed, and after CO₂ evacuation, the incisions were sutured. The procedure was completed without complications.

### 2.4 Outcome measures

Clinical data were collected for both groups, including sex, body weight, age, duration of abdominal pain before admission, appendicitis type, operative time, intraoperative blood loss (Blood loss was estimated based on suction volume and surgical gauze assessment), time to first postoperative flatus, length of postoperative hospital stay, postoperative complications (including incision infection, abdominal pain, intestinal obstruction, stump inflammation, and fecal leakage), ultrasonographic findings at 3 months postoperatively, and postoperative levels of C-reactive protein (CRP) and interleukin-6 (IL-6).

### 2.5 Diagnostic and evaluation criteria

The diagnostic criteria for postoperative complications were predefined and consistently applied to both groups:

2.5.1 Postoperative abdominal pain was evaluated using the visual analog scale (VAS). Clinically significant pain was defined as persistent or recurrent abdominal pain with a VAS score ≥ 4 lasting more than 48 hours post-surgery, not attributable to routine recovery.

2.5.2 Subacute intestinal obstruction was diagnosed with symptoms such as intermittent abdominal pain, distension, nausea, or vomiting, combined with imaging findings showing dilated bowel loops or delayed transit, without acute strangulation or complete obstruction.

2.5.3 Stump inflammation was defined as localized inflammatory changes at the appendiceal stump, observed via ultrasonography or CT, including wall thickening, increased echogenicity, or fluid collection, along with elevated inflammatory markers or localized tenderness, without stump leakage.

2.5.4 Incision infection was diagnosed based on standard criteria, including redness, swelling, warmth, pain, or purulent discharge at the incision site, with or without positive bacterial culture.

2.5.5 Fecal leakage was defined as the presence of fecal material in the peritoneal cavity (confirmed intraoperatively or by imaging) or drainage of enteric content through an abdominal drain, accompanied by peritonitis signs.

2.5.6 Ultrasonographic evaluation of adhesions: No adhesion was defined as an inter-tissue space width ≥2 mm and absence of strip-like bands. Mild adhesion was defined as 1–2 mm space width and 1–3 strip-like bands (≤1 mm thick). Dense adhesion was defined as space width <1 mm or obliteration, with ≥4 strip-like bands (>1 mm thick or reticular fusion).

### 2.6 Statistical analysis

Data were organized and analyzed using SPSS version 26.0 (IBM Corp., Armonk, NY, USA).Continuous variables were assessed for normality using the Kolmogorov–Smirnov test. Variables that did not conform to a normal distribution were expressed as the median (interquartile range), and intergroup comparisons were performed using the Mann–Whitney U test. Categorical variables were presented as frequencies (percentages), and comparisons between groups were conducted using the chi-square test or Fisher's exact test, as appropriate. A two-sided P value of <0.05 was considered statistically significant. Because this was a retrospective exploratory study, no *a priori* power calculation was performed for rare postoperative complications. Therefore, comparisons involving low-frequency events should be interpreted cautiously and considered hypothesis-generating rather than definitive. For rare categorical outcomes with low expected cell counts, Fisher's exact test was considered more appropriate and was used where applicable.

## 3 Results

### 3.1 Patient enrollment and group allocation

A total of 133 pediatric patients were included in the final analysis, including 70 in the absorbable-clip group and 63 in the Hem-o-lok clip group. All patients successfully underwent the intended procedure. In the Hem-o-lok clip group, two patients developed recurrent postoperative abdominal pain and subacute intestinal obstruction, requiring reoperation at 8 and 10 weeks after appendectomy, respectively. Both of these patients had suppurative appendicitis at the initial operation. During re-exploration, dense adhesions around the Hem-o-lok clips in the ileocecal region were the most prominent findings, with no evidence of stump inflammation or other bowel pathology. As no silk ligature was used in the Hem-o-lok group, silk-related foreign-body reactions were excluded in these cases.

### 3.2 Comparison of baseline characteristics between the two groups

There were no statistically significant differences between the two groups in sex, age, appendicitis type, duration of abdominal pain, or body weight (*P* > 0.05).Detailed baseline characteristics are presented in Table 1.

**Table 1:** Comparison of baseline characteristics between the two groups .

Variables	Absorbable clip group(*n* = 70)	Hem-o-lok clip group(*n* = 63)	*χ*^2^*/z* value	*P* value
Sex, *n*(%)			0.971^b^	0.325
Male	49 (70.0)	39 (61.9)		
Female	21 (30.0)	24 (38.1)		
Age (years), *M*(*P*_25_, *P*_75_)	7.50 (5.75, 11.00)	8.00 (6.00, 10.00)	−0.143^a^	0.887
Type of appendicitis, n (%)			-^c^	0.532
Perforated	18 (25.7)	18 (28.6)		
Simple	14 (20.0)	13 (20.6)		
Suppurative	36 (51.4)	27 (42.9)		
Gangrenous	2 (2.9)	5 (7.9)		
Duration of abdominal pain (h), *M*(*P*_25_, *P*_75_)	24.00 (12.00, 48.00)	24.00 (12.00, 48.00)	−0.028^a^	0.978
Body weight (kg), *M*(*P*_25_, *P*_75_)	26.50 (22.00, 42.25)	30.00 (22.00, 41.00)	−0.165^a^	0.869

a Mann–Whitney U test.

b Chi-square test.

c Independent-samples *t* test.

### 3.3 Comparison of perioperative outcomes between the two groups

There were no statistically significant differences between the two groups in abdominal drain placement, time to drain removal, intraoperative blood loss, time to first postoperative flatus, or postoperative length of hospital stay (*P* > 0.05).The operative time was significantly shorter in the Hem-o-lok clip group than in the absorbable clip group (*P* < 0.05).These results are summarized in Table 2.

**Table 2:** Comparison of perioperative outcomes between the two groups.

Variable	Absorbable clip group(*n* = 70)	Hem-o-lok clip group(*n* = 63)	*χ*^2^*/z/t* value	*P value*
Operative time (min), *M*(*P*_25_, *P*_75_)	45.00 (30.00, 52.00)	34.00 (25.00, 47.00)	−2.254^a^	0.024
Intraoperative blood loss (mL), *M*(*P*_25_, *P*_75_)	2.00 (1.00, 3.50)	2.00 (1.00, 5.00)	−0.049^a^	0.961
Time to first postoperative flatus(days), *M*(*P*_25_, *P*_75_)	2.00 (2.00, 2.00)	2.00 (2.00, 2.00)	−0.828^a^	0.408
Postoperative hospital stay (days), *M*(*P*_25_, *P*_75_)	6.00 (5.00, 7.00)	6.00 (5.00, 8.00)	−1.505^a^	0.132
Abdominal drain placement, *n*(%)	4 (5.7)	6 (9.5)	0.692^b^	0.405
Time to drain removal (days), $\bar{x}\pm s$	2.75 ± 0.96	3.00 ± 0.89	−0.422^c^	0.684

a Mann–Whitney U test.

b Chi-square test.

c Independent-samples *t* test.

### 3.4 Comparison of postoperative complications between the two groups

**Figure 4:**
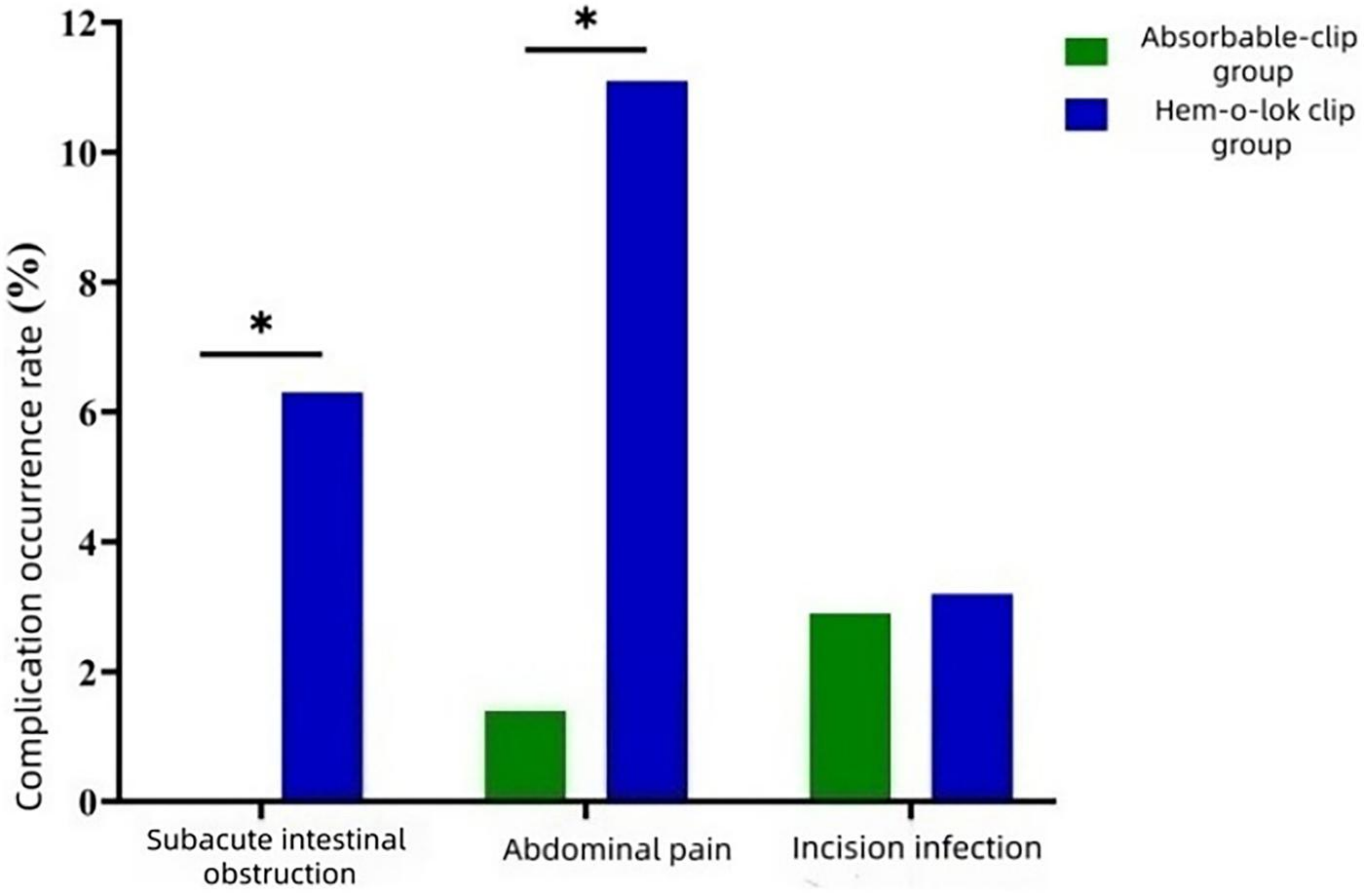
Comparison of postoperative complications between the two groups; *P < 0.05.

No significant differences were observed between the two groups in the incidence of incision infection, stump inflammation, or fecal leakage (*P* > 0.05).The proportions of subacute intestinal obstruction and abdominal pain were significantly higher in the Hem-o-lok clip group than in the absorbable clip group (*P* < 0.05).These findings are presented in Table 3 and Figure 4.

**Table 3:** Comparison of postoperative complications between the two groups, *n* (%).

Variable	Absorbable clip group(*n* = 70)	Hem-o-lok clip group(*n* = 63)	*χ*^2^ value	*P* value
Postoperative complications				
Subacute intestinal obstruction	0 (0)	4 (6.3)	-	0.048
Abdominal pain	1 (1.4)	7 (11.1)	-	0.027
Incision infection	2 (2.9)	2 (3.2)	-	1.000
Stump inflammation	0 (0)	0 (0)	-	-
Fecal leakage	0 (0)	0 (0)	-	-

### 3.5 Comparison of ultrasonographic findings of the appendiceal stump between the two groups

**Figure 5:**
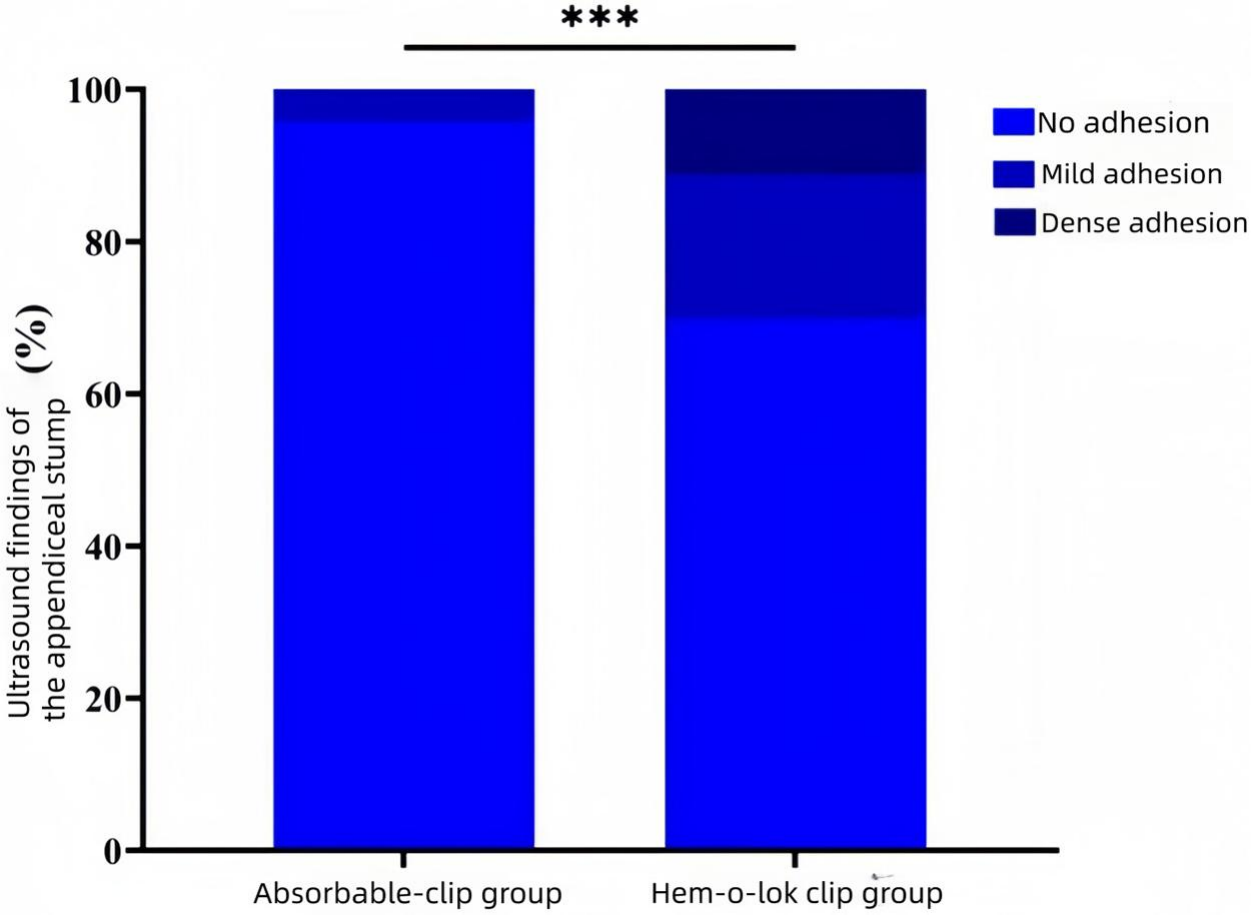
Comparison of ultrasound findings of the appendiceal stump between the two groups; ***P < 0.001.

A statistically significant difference was observed between the two groups with respect to ultrasonographic findings of the appendiceal stump (*P* < 0.05).Pairwise comparisons showed that the proportions of “mild adhesions” and “dense adhesions” on ultrasonography were significantly higher in the Hem-o-lok clip group than in the absorbable clip group (*P* < 0.05).Conversely, the proportion of patients with “no adhesions” was significantly lower in the Hem-o-lok clip group than in the absorbable clip group (*P* < 0.05).These results are shown in Table 4 and Figure 5.

**Table 4:** Comparison of ultrasound findings of the appendiceal stump between the two groups, *n* (%).

Variables	Absorbable clip group(*n* = 70)	Hem-o-lok clip group(*n* = 63)	*χ*^2^ value	*P* value
Ultrasound findings of the appendiceal stump			-	<0.001
No adhesion	67 (95.7)	44 (69.8)^a^		
Mild adhesion	3 (4.3)	12 (19.0)^a^		
Dense adhesion	0 (0)	7 (11.2)^a^		

Compared with the absorbable clip group.

a *P* < 0.05.

### 3.6 Comparison of postoperative CRP and Il-6 levels between the two groups

**Figure 6:**
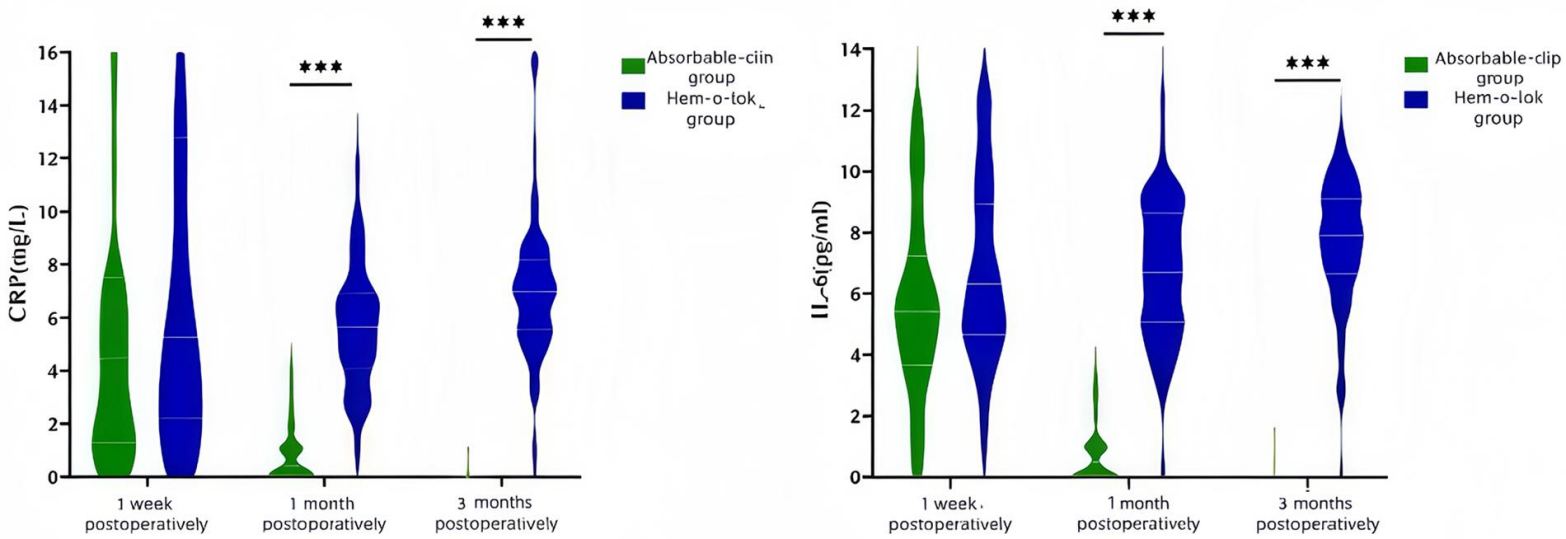
Comparison of postoperative CRP and IL-6 levels between the two groups; ***P < 0.001.

No statistically significant differences were observed between the two groups in C-reactive protein (CRP) or interleukin-6 (IL-6) levels at 1 week postoperatively (*P* > 0.05).At 1 month and 3 months postoperatively, CRP and IL-6 levels were significantly higher in the Hem-o-lok clip group than in the absorbable clip group (*P* < 0.05). These results are presented in Table 5 and Figure 6.

**Table 5:** Comparison of postoperative CRP and IL-6 levels between the two groups, *M*(*P*_25_, *P*_75_).

Variables	Absorbable clip group (*n* = 70)	Hem-o-lok clip group (*n* = 63)	*Z* value	*P* value
CRP(mg/L)				
1 week postoperatively	4.49 (1.26, 7.53)	5.24 (2.21, 12.77)	−1.498	0.134
1 month postoperatively	0.42 (0.05, 1.30)	5.64 (4.09, 6.90)	−9.348	<0.001
3 months postoperatively	0.01 (0.01, 0.05)	6.98 (5.55, 8.14)	−9.783	<0.001
IL-6(pg/mL)				
1 week postoperatively	5.41 (3.63, 7.23)	6.30 (4.64, 8.90)	−1.841	0.066
1 month postoperatively	0.41 (0.03, 1.06)	6.69 (5.07, 8.64)	−9.723	<0.001
3 months postoperatively	0.01 (0.01, 0.05)	7.89 (6.66, 9.10)	−9.717	<0.001

## 4 Discussion

Single-incision laparoscopic appendectomy has become an attractive option for pediatric appendicitis because of its minimal invasiveness and favorable cosmetic results (9). However, limited operative space and instrument interference increase the technical demands of stump closure and mesoappendiceal management. Although Hem-o-lok clips are widely used because of their convenience, concern remains regarding tissue reaction and adhesion formation caused by retained nonabsorbable foreign material. In this study, we evaluated a modified single-incision two-port laparoscopic suspension technique combined with single-instrument knotting and absorbable clips. The findings suggested that this approach maintained operative feasibility while reducing postoperative complications and tissue inflammatory responses.

### 4.1 Rationale and operative feasibility of the modified surgical technique

Single-incision laparoscopic surgery is technically challenging because of limited operative space and instrument interference (10). In the present study, the two-port configuration and suspension technique helped improve exposure of the appendiceal base and mesoappendix while preserving the cosmetic advantage of a periumbilical approach. This arrangement reduced instrument collision and allowed the procedure to be completed with acceptable operative efficiency.

From a perioperative perspective, the absorbable clip group had a slightly longer operative time than the Hem-o-lok group, whereas other perioperative outcomes were comparable. These findings suggested that the modified technique did not substantially increase procedural complexity or surgical risk. The longer operative time was likely related to the learning curve of single-instrument knotting, and this difference decreased as surgical proficiency improved.

An important feature of this technique is the dual-closure strategy, which combines ligation with absorbable clip reinforcement. This approach may improve stump security by reducing reliance on a single closure method. In addition, compared with exclusive use of Hem-o-lok clips, the modified technique reduced the number of retained nonabsorbable clips, which may help decrease foreign-body-related inflammation and adhesion formation. Absorbable clips may also be better suited to the constrained working space of single-incision surgery because of their more adaptable clamping characteristics.

Endoloop ligation remains another commonly used option for appendiceal stump closure (11). Although it is considered safe and cost-effective, its effectiveness depends on circumferential suture tension and may be less reliable when the appendiceal base is edematous or friable (12, 13). In contrast, the combined ligation-and-clip strategy used in this study provided additional mechanical support. Previous studies have also suggested that polymeric clips and absorbable materials may offer safety profiles comparable or superior to those of endoloops in selected settings (14).

### 4.2 Mechanistic analysis of advantages in complication control

A key finding of the present study was that the overall incidence of complications was significantly lower in the absorbable clip group than in the Hem-o-lok clip group, with particularly pronounced differences observed in adverse events such as postoperative abdominal pain and subacute intestinal obstruction. These results directly highlight the clinical importance and advantages of minimizing residual foreign materials.

Postoperative abdominal pain and subacute intestinal obstruction may have resulted from the persistent foreign-body response caused by Hem-o-lok clips, which are nonabsorbable polycarbonate implants. Hem-o-lok clips are polymer-based devices made of nonconductive, inert, and nonabsorbable materials (15). They are commonly used in laparoscopic procedures such as cholecystectomy, colorectal resection, splenectomy, gastrectomy, and appendectomy, offering advantages like anti-slippage design, reliable fixation, effective ligation, and ease of use (16). However, as nonabsorbable foreign bodies, they may trigger host rejection and inflammation upon implantation (17). This process involves macrophage accumulation, cytokine release, fibroblast proliferation, and fibrous tissue encapsulation, leading to adhesions between the bowel, mesentery, and appendiceal stump (18). The micro-protruding surface of Hem-o-lok clips further promotes adhesion formation (19). Studies have shown a high incidence of adhesions with retained Hem-o-lok clips, which may lead to adhesive intestinal obstruction in severe cases (20). In contrast, absorbable clips used in this study, made from polyglycolic acid (PGA) or polylactic acid (PLA), degrade gradually *in vivo* into carbon dioxide and water, eventually being absorbed by the body (21). The degradation period typically lasts about 3 months, coinciding with the tissue healing phase. In this study, the absorbable clip group had a significantly lower incidence of intra-abdominal adhesions (4.3%) compared to the Hem-o-lok clip group (30.2%). Ultrasonographic evaluation also showed less peristump tissue thickening in the absorbable clip group. Two pediatric patients in the Hem-o-lok clip group required reoperation due to persistent pain and intestinal obstruction. Dense adhesions around the Hem-o-lok clips were found during surgery, and pain was markedly alleviated after clip removal, providing clinical evidence that the absence of foreign material plays a key role in reducing adhesions. Previous studies have similarly indicated that while Hem-o-lok clips are biocompatible and widely used, their nonabsorbable nature may increase the risk of postoperative complications, especially when retained near peristaltic bowel loops. Therefore, absorbable materials may be preferred for appendiceal stump closure in laparoscopic appendectomy to reduce such risks (22, 23).

### 4.3 Quantitative validation of tissue response intensity

Quantitative assessments of serum inflammatory markers and imaging findings provided objective evidence supporting the superior tissue compatibility of the modified surgical technique. C-reactive protein (CRP) and interleukin-6 (IL-6) are sensitive indicators of systemic inflammation, often elevated due to surgical trauma and foreign-body stimulation (24). At 1 week postoperatively, no significant differences in CRP or IL-6 levels were observed between the absorbable clip and Hem-o-lok clip groups, likely due to incomplete resolution of postoperative inflammation during the early recovery phase. However, at 1 and 3 months postoperatively, inflammatory markers remained higher in the Hem-o-lok clip group than in the absorbable clip group. This difference should be interpreted cautiously and not solely based on statistical significance. CRP, as a nonspecific marker, can be influenced by factors such as infections, immune status, or unrelated inflammatory conditions at 3 months post-surgery, making it unreliable as the sole indicator of local foreign-body reaction. For instance, the median CRP value of 6.98 mg/L in the Hem-o-lok group at 3 months suggests persistent low-grade inflammation or foreign-body response, but the absolute elevation is borderline. Therefore, its clinical relevance should be considered alongside symptoms, imaging, and longer-term follow-up, rather than as definitive evidence of clinically significant inflammation. In contrast, inflammatory markers in the absorbable clip group rapidly declined and approached baseline values.

Based on ultrasonographic findings, a higher proportion of patients in the Hem-o-lok clip group exhibited peristump tissue thickening and heterogeneous echogenicity at 3 months postoperatively. In some cases, a circumferential hyperechoic ring surrounding the Hem-o-lok clip was observed, suggesting the formation of fibrous tissue encapsulation. In contrast, the absorbable clip group demonstrated peristump tissue morphology more closely resembling normal intra-abdominal structures, with no obvious abnormal echogenicity. These findings suggestded an association between absorbable clip use and a milder postoperative inflammatory profile. However, because this was a retrospective non-randomized study, the observed differences in CRP and IL-6 cannot be attributed solely to clip type, and potential confounding factors, including local disease severity, tissue handling, and other postoperative inflammatory influences, cannot be completely excluded. These imaging results were consistent with clinical outcomes, as patients in the absorbable clip group experienced fewer episodes of postoperative abdominal pain and intestinal obstruction, highlighting the positive impact of a reduced inflammatory response on postoperative quality of life.

### 4.4 Clinical applicability and translational value of the surgical technique

The modified technique may be particularly useful in pediatric patients in whom cosmetic outcome and reduction of postoperative adhesions are important. It may also be advantageous in cases with an edematous or fragile appendiceal base, where additional stump security is desirable. The relatively short learning curve observed in our practice suggested that surgeons already experienced in laparoscopic appendectomy could become familiar with the suspension and single-instrument knotting technique after targeted training.

From a practical perspective, the per-use cost of absorbable clips was comparable to that of Hem-o-lok clips. Although the modified technique required slightly longer operative time, it may reduce secondary treatment costs associated with postoperative complications, which could improve long-term cost-effectiveness.

### 4.5 Study limitations and future directions

This study has several limitations. First, because it was retrospective and non-randomized, selection bias and indication bias could not be completely excluded. Second, the sample size was limited, especially for rare complications such as subacute intestinal obstruction, and the findings should therefore be considered preliminary. Third, the follow-up period was only 3 months, which was sufficient for evaluating early inflammatory response but insufficient for assessing long-term adhesion-related outcomes, chronic abdominal symptoms, or delayed bowel obstruction. Longer follow-up will be needed to define the long-term safety profile of the two techniques.

In addition, the modified single-site two-port technique has technical limitations. In cases of severe local inflammation, extensive adhesions, friable appendiceal bases, or inadequate exposure of the ileocecal region, continuation through a single-site two-port approach may not be safe. Under such circumstances, timely conversion to a conventional three-port laparoscopic approach or open surgery is appropriate and should be regarded as prudent surgical judgment rather than procedural failure (25–27).

Future studies should include large multicenter prospective trials with longer follow-up. Further evaluation in adult and elderly populations is also warranted. In addition, clip-free or ligation-based strategies may represent a useful direction for future optimization of appendiceal stump management.

## 5 Conclusion

The modified single-incision two-port laparoscopic suspension appendectomy combined with single-handed knotting overcame some technical limitations of single-incision surgery through a dual-port configuration and suspension-assisted exposure. By replacing Hem-o-lok clip closure with a strategy integrating absorbable clips and single-handed knotting, this technique achieved three main objectives: minimal invasiveness, reduced retention of nonabsorbable polymer clips, and secure stump closure. Clinical evidence from the present study showed that, while maintaining operative efficiency and the benefits of minimally invasive surgery, this approach reduced postoperative complications such as abdominal pain and intra-abdominal adhesions, attenuated tissue inflammatory responses, and improved postoperative recovery quality. Compared with conventional Hem-o-lok clip closure, the modified technique appeared to align more closely with the principles of precision surgery and enhanced recovery after surgery (ERAS), suggesting potential value for broader clinical application. Given the retrospective design, non-randomized group allocation, limited sample size for rare events, and relatively short follow-up period, these findings should be considered preliminary and hypothesis-generating. Larger prospective studies with at least 12 months of follow-up are warranted to confirm the long-term safety and clinical value of this technique.

## Data Availability

The original contributions presented in the study are included in the article/Supplementary Material, further inquiries can be directed to the corresponding author.
